# A Recombinase Polymerase Amplification and Lateral Flow Strip Combined Method That Detects *Salmonella enterica* Serotype Typhimurium With No Worry of Primer-Dependent Artifacts

**DOI:** 10.3389/fmicb.2020.01015

**Published:** 2020-06-23

**Authors:** Huahua Wu, Panpan Zhao, Xiaohan Yang, Juan Li, Jingyu Zhang, Xun Zhang, Zihan Zeng, Jingquan Dong, Song Gao, Chen Lu

**Affiliations:** ^1^Jiangsu Key Laboratory of Marine Biological Resources and Environment, Jiangsu Key Laboratory of Marine Pharmaceutical Compound Screening, Co-Innovation Center of Jiangsu Marine Bio-industry Technology, Jiangsu Ocean University, Lianyungang, China; ^2^Key Laboratory of Zoonosis Research by Ministry of Education, College of Veterinary Medicine, Jilin University, Changchun, China; ^3^Wuhan Institute for Food and Cosmetic Control, Wuhan, China; ^4^Department of Animal Science, College of Wildlife and Protected Area, Northeast Forestry University, Harbin, China; ^5^School of Pharmacy, Jiangsu Ocean University, Lianyungang, China

**Keywords:** recombinase polymerase amplification, lateral flow strip, primer-dependent artifacts, *Salmonella enterica* serotype Typhimurium, false positive

## Abstract

On-site detection demands are quickly increasing to control foodborne pathogenic bacteria along with the long food supply chains. Combining the isothermal recombinase polymerase amplification (RPA) with lateral flow strips (LFSs) is a promising molecular detection approach for the short reaction time, low isothermal condition, and simple and “instrument-free” procedure. However, the method comes with a non-negligible intrinsic risk of the primer-dependent artifacts. In this study, with an important foodborne pathogenic bacterium *Salmonella enterica* serotype Typhimurium (*S.* Typhimurium) as the model, system measures including the careful selection of primers targeting unique virulence genes, use of a probe in the RPA reaction, introducing base substitutions with specific guidelines in the primer and probe sequences, and analyzing and screening the primer–probe complex formation were taken to eliminate the primer-dependent artifacts. The measures were strictly tested for the efficacy, and the standardized method was able to specifically detect *S. typhimurium* within 30 min at 42°C without any interference of probe–primer signals. The established RPA-LFS method shared high sensitivity with the detection limit of 1 CFU/μl of unpurified culture. Our study provided practical measures for the prevention of false positive signals from primer–dimers or primer–probe complexes when using the RPA–LFS method in pathogen detections, and also established a readily applicable method for *S.* Typhimurium detection.

## Introduction

Effective detection and close monitoring of foodborne pathogenic bacteria are essential for food safety management and public health (Zhao et al., 2014; Sekse et al., 2017; Zhang et al., 2018). Commonly seen foodborne pathogenic bacteria include *Salmonella*, *Staphylococcus aureus*, *Escherichia coli* O157, *Listeria monocytogenes*, *Bacillus cereus*, *Campylobacter jejuni*, and *Vibrio parahaemolyticus* and the control of contamination by them in food supply chains is required by the World Health Organization and administrative organizations in many countries (Hendriksen et al., 2011; Abebe et al., 2017). With the fast growth of global economics of this era, food supply chains are becoming increasingly long, and cooperations among food-producing enterprises are far more complicated (Newell et al., 2010; Hathaway, 2013). This situation has put a big challenge on the food safety management and public health, and bacteria detection technologies with faster speed, higher accuracy, simplicity, and convenience are demanded all the time.

Biochemical assays and molecular approaches are two major technologies for detecting bacteria and have made great contributions to the detection, identification, and control of spreading of foodborne pathogens (Umesha and Manukumar, 2018; Moezi et al., 2019). With this being said, however, biochemical assays are usually associated with the bacterium cultivation, morphological observation, and serologic confirmation tests, which are laborious and time-consuming and do not meet, nowadays, the needs for rapid detection (Zhao et al., 2014). Molecular approaches based on polymerase chain reaction (PCR) have been extensively applied to pathogenic bacteria detection in the past decades. These PCR-based methods can give detection results within several hours and have become the major methods in pathogen detection applications (Salazar et al., 2015; Zhang et al., 2018; Vinayaka et al., 2019), but still, the dependency on complicated thermal cycling machines and trained personnel limited their use for on-site detection or under resource-limited settings (Dai et al., 2019). Recent development of isothermal amplification technologies, including loop-mediated isothermal amplification (LAMP) and recombinase polymerase amplification (RPA), are promising solutions because these technologies avoid the use of expensive and complicated thermal cycling machines and could be conducted by people with limited training (Li et al., 2017; Wu et al., 2019).

RPA has been considered more useful for pathogen detection applications over LAMP for its improved amplification specificity (Daher et al., 2016; Dai et al., 2019). This technology uses the recombinase activity of the enzyme to open the double strands of DNA molecules and the strand-displacing activity to amplify DNA targets. The amplification could be finished within 20–30 min in a temperature range of 37°–42°C (Daher et al., 2016). Advantages of rapidness and near-ordinary reaction temperature make the RPA technology quite convenient for pathogen detection applications.

**FIGURE 1:**
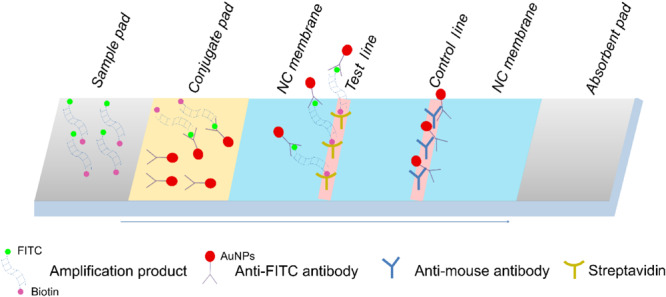
Schematic representation of the lateral flow strip (LFS) working principle. The names of the segments of the strip were indicated on the top of the strip drawing. The sample pad is indicated by the gray parallelogram on the left, the absorbent pad is indicated by the gray parallelogram on the right, and the conjugate pad is shown in yellow. The liquid migration direction is indicated by an arrow. Molecules could be trapped by the materials on the test line, and the control line is indicated by different shapes. Shapes and their representing molecules are listed at the bottom of the image.

The end-point readout of the isothermally amplified DNA target can be conducted with gel electrophoresis or fluorescent nucleic acid staining, and chemical labeling of the RPA reaction combined with the use of lateral flow strips (LFS) has made “instrument-free” signal readouts possible (Qi et al., 2018). By using gold nanoparticles (AuNPs) specifically interacting with the labeled isothermal amplification products, colored signals are observed with the naked eye (Figure 1; Du et al., 2018b). Briefly, the amplification product was modified with FITC and biotin at the two ends, and the anti-FITC antibody from mouse was functionalized with AuNPs. After the amplification products are loaded onto the sample pad, they migrate through the conjugate pad and are bound with the anti-FITC AuNPs. When these amplification products reach the test line that coated with streptavidin, they are trapped to show the red color. The anti-FITC antibody molecules not bound to the amplification product continue migration to the control line to validate the strip test. Combining LFS with RPA enables pathogen detections without any special equipment. Promising results have been reported for detection of *S. aureus*, *Salmonella*, and *L. monocytogenes* (Liu et al., 2017; Du et al., 2018a, b).

Nevertheless, primer-dependent artifacts should be considered as an important intrinsic risk when using LFS with RPA. Primer–dimers are difficult to eliminate in DNA amplification reactions (Poritz and Ririe, 2014; Meagher et al., 2018). PCR-based methods work at high annealing temperature to provide high specificity. In contrast, primer binding in RPA is done at room temperature and may produce false signal. On the other hand, the LFS detection does not have the ability to distinguish the size of the molecule that gives the signal. Because the sensitivity the RPA–LFS combined method is very high, special measures have to be taken to prevent the interference of signal from probe–dimers.

In this study, special measures including the use of a probe in the RPA reaction and introducing base substitutions in the primer and probe sequences were taken to eliminate the primer-dependent artifacts in the RPA–LFS combined method. An important foodborne pathogenic bacterium, *Salmonella enterica* serotype Typhimurium (*S.* Typhimurium), was used as the model for the method development. The measures were strictly tested for the efficacy on eliminating the primer-dependent artifacts, and the standardized RPA–LFS combined method was able to specifically detect 1 CFU of unpurified *S.* Typhimurium within 30 min at 42°C without any interference of probe–primer signals. Our study provided practical measures for eliminating primer-dependent artifacts in the detection of pathogenic bacteria with the RPA–LFS combined method and also established a rapid, accurate, and simple detection method that is readily applicable to the *S.* Typhimurium detection.

## Materials and Methods

### Bacterial Strains

The thermal inactivated standard strains of *S.* Typhimurium (ATCC14028), *S. aureus*, *E. coli* O157, *L. monocytogenes*, *B. cereus*, and *V. parahaemolyticus* at a concentration of 10^7^ colony-forming units (CFU)/ml in LB medium were kindly given by the Wuhan Institute for Food and Cosmetics control (Wuhan, China). For qPCR or RPA reactions, the inactivated cultures were heat treated at 100°C for 10 min before serving as the templates.

### Design of RPA Primers

RPA primers were designed on the NCBI Primer-BLAST website^1^ according to sequences of virulence genes of *invA* and *invE* from *S.* Typhimurium genome (GenBank Acc. No. CP034479.1). The database was set as Refseq representative genomes. The product size, primer size, and primer GC content were set as 150–300 bp, 28–35 nt, and 30–70%, respectively. The max self-complementarity and pair complementarity were set 3 bp at both 5′ and 3′ ends. Other parameters were set as default.

### RPA Procedure and Electrophoresis

RPA reactions were performed according to the manufacturer’s instructions of the TwistAmp^®^ Liquid DNA Amplification Kit (TwistDx, Inc., Maidenhead, United Kingdom). A 50-μl reaction contained 46.5 μl of reaction mixture, 1 μl of thermal inactivated bacteria solution, and 2.5 μl of 280 mM magnesium acetate. The reaction contained 25 μl of 2 × Reaction buffer, 5 μl of 10 × Basic e-mix, 2.5 μl of 20 × core mix, 2.4 μl of 10 μM forward primer, 2.4 μl of 10 μM reverse primer, and 9.2 μl of distilled water. Magnesium acetate, 2.5 μl of 280 mM, and 1 μl of the template were added to the lid of the reaction tube. After brief centrifugation, the reaction mixture was immediately incubated at 37°C for 30 min. The RPA amplification products were purified using PCR Cleaning Kit (Monad Biotech, Co., Ltd., Wuhan, China) and electrophoresed on a 2% agarose gel.

### RPA–LFS Procedure With Forward and Reverse Primers

The forward and reverse primers were modified with fluorescein isothiocyanate (FITC) and biotin at the 5**′** ends, respectively (General Biosystems, Co. Ltd., Anhui, China). RPA reactions were performed according to the manufacturer’s instructions of the TwistAmp^®^ Liquid DNA Amplification Kit (TwistDx, Inc., Maidenhead, United Kingdom). Five microliters of the amplification products were used for LFS (Ustar Biotech, Ltd., Hangzhou, China) detection. The amplification products were mixed with 95 μl of sample buffer (Ustar Biotech), and the stick of LFS was inserted into the mixture for 3 min and then for visual reading.

### Design of Probes

The probes were designed using the Primer Premier 5.0 software (Premier Biosoft International, Palo Alto, CA, United States). The size, GC content, and Tm of the probe were set as 46–53 nt, 20–80%, and 57–80°C, respectively. The max hairpin and primer–dimer was set as less than four bonds within six bases of the 3′ end. The max poly-X was set as 6. Other parameters were set as default.

### RPA–LFS Procedure With Primers and a Probe

The reverse primers and probes were modified at the 5′ ends with biotin and fluorescein isothiocyanate (FITC), respectively (General Biosystems). The 3′ end of the probe was labeled with a C3-spacer (SpC3) that could block the amplification, and a tetrahydrofuran ([THF]) site was put in the middle of the probe for nfo enzyme cleavage. The nfo enzyme would function after bases flank the [THF] site pairing with the other strand and free the 3′ end of the probe for elongation. RPA reactions were setup according to the manufacturer’s instructions of TwistAmp^®^ DNA Amplification nfo Kit (TwistDx). The reaction contained 46.5 μl of reaction mixture, 1 μl of bacteria solution, and 2.5 μl of 280 mM magnesium acetate. The reaction mixture consisted of 29.5 μl of Rehydration Buffer, 2.1 μl of 10 μM forward primer, 2.1 μl of 10 μM reverse primer, 0.6 μl of 10 μM probe, and 12.2 μl of distilled water. To initiate the reaction, 1 μl of template and 2.5 μl of 280 mM magnesium acetate was added into the mixture. After brief centrifugation, the reaction mixture was immediately incubated at 40°C for 30 min. Two microliters of the amplification products were used for LFS (Ustar Biotech) detection. The amplification products were added to the sample pad of LFS, and the stick of LFS was inserted into 100 μl of the sample buffer (Ustar Biotech) for 3 min and then for visual reading.

### Preparation of Artificially *S.* Typhimurium Contaminated Samples

Sterilized pure milk was purchased from a local supermarket. The milk samples were divided equally into 50 pieces and 8 of them were artificially contaminated with *S.* Typhimurium culture to a final concentration of 10^2^ CFU/μl. Fifty samples were randomly numbered and subjected for detection of *S.* Typhimurium with both RPA–LFS and qPCR methods.

### Quantitative PCR (qPCR)

A pair of specific primers (F-GTGAAATTATCGCCAC GTTCGGGCAA and R-TCATCGCACCGTCAAAGGAACC) targeting to the *invA* gene of *S.* Typhimurium was used for qPCR (Kumar et al., 2010). The qPCR reactions were performed according to the manufacturer’s instructions of the MonAmp^TM^ SYBR^®^ Green qPCR Mix (Monad Biotech, Ltd., Wuhan, China) on a LightCycler^®^ 480II qPCR Instrument (Roche, Switzerland). The reaction mixture contained 10 μl of MonAmp^TM^ SYBR^®^ Green qPCR Mix (Monad Biotech), 0.4 μl of each primer (10 μM), 1 μl of the sample, and 8.2 μl of distilled water. The cycling program was 95°C for 30 s, followed by 40 cycles of 95°C for 10 s, then 60°C for 30 s. The melting curve analysis was set as default.

## Results

### Primer Design and Screening

**FIGURE 2:**
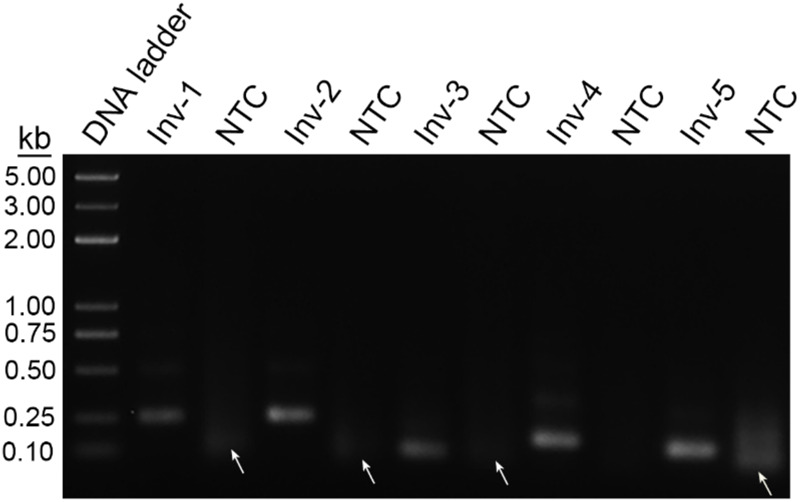
Screening of primer pairs by recombinase polymerase amplification (RPA) amplification performance. The agarose gel image shows the amplification results of the primer pairs targeting virulence genes of *InvA* and *InvE*. Each primer pair name is indicated on the top of each lane. The NTC lane immediately after is the no-template control of the respective primer pair. The band sizes of the DNA ladder are shown on the left. Primer dimers are indicated by white arrows.

Initial sequence screening for potential targeting sites of the primers focused on the *invA* and *invE* virulence genes of *S.* Typhimurium, which had been widely used as the biomarker for molecular detection of this pathogen (Stone et al., 1994; Kasturi and Drgon, 2017). Using NCBI Primer-BLAST, five potential primer pairs were obtained that met the following parameter settings: (1) the primer pair should only target the species of interest (*S.* Typhimurium); (2) the primer pair should have less than five consecutive bases (and less than three if located at the 3′ end) pairing each other (Table 1). The five primer pairs were screened for amplification performance in the RPA reaction (Figure 2). All the five primer pairs showed amplification of the target fragments; however, four of them showed obvious primer–dimer bands when the DNA templates were not present. Only the primer pair Inv-4 produced the specific amplification band while showing no detectable primer–dimer band on the agarose gel.

**TABLE 1:** Primer sequences and targeting areas.

**Name**	**Sequence (5′–3′)**	**Primer length (nt)**	**Gene name**	**Amplicon (bp)**	**Site in genome (GenBank Acc. No. CP034479.1)**
Inv-1	F: 5**′**-GATCATCACCATTAGTACCAGAATCAGTAA-3**′**	30	*InvA*	247	998061. 998090
	R: 5**′**-ATTTTATCAAGTATGTAAAGCCATACCCTC-3**′**	30	*InvE*		997844. 997873
Inv-2	F: 5**′**-ATCATCACCATTAGTACCAGAATCAGTAA-3**′**	29	*InvA*	245	998061. 998089
	R: 5**′**-TTTTATCAAGTATGTAAAGCCATACCCTC-3**′**	29	*InvE*		997845. 997873
Inv-3	F: 5**′**-GTTGAAAAACTGAGGATTCTGTCAATGTAG-3**′**	30	*InvA*	111	998186. 998215
	R: 5**′**-CATTCCATTACCTACCTATCTGGTTGATTT-3**′**	30			998105. 998134
Inv-4	F: 5**′**-CTACAAGCATGAAATGGCAGAACAGCGTCG-3**′**	30	*InvE*	188	997943. 997972
	R: 5**′**-CAACCAGATAGGTAGGTAATGGAATGACGA-3**′**	30	*InvA*		998101. 998130
Inv-5	F: 5**′**-CCTTTACTGGTTTTAGGTTTGGCGG-3**′**	25	*InvA*	162	998989. 999013
	R: 5**′**-ATTTGTATTGGTTGTTACGGCTATTTTGAC-3**′**	30			998852. 998881

*F, forward primer; R, reverse primer.*

**FIGURE 3:**
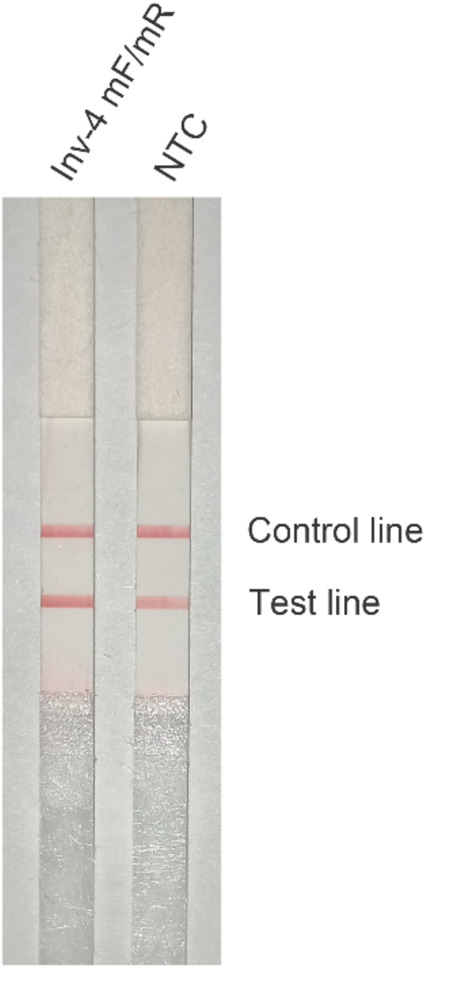
Test of the primer pair Inv-4 on RPA–LFS. The primer pair Inv-4 is labeled with fluorescein isothiocyanate (FITC) and biotin for RPA–LFS detection. The NTC lane is the no-template control. The original primer pair Inv-4 gives false-positive signals on the RPA–LFS system. The test lines and control lines are marked on the right side of the picture. The reactions were performed at 37°C for 30 min.

The primer pair Inv-4 was labeled with FITC and biotin for LFS detection of the amplification products (Table 2). The LFS result indicated that, even though the RPA amplification did not show any primer–dimer band on the agarose gel, false positive signal was still present on the strip (Figure 3). The band density of the test line from the amplification without DNA template was comparable to that from the normal RPA reaction.

**TABLE 2:** Modification of primer pair Inv-4 with chemical labels.

**Name**	**Sequence (5′–3′)**	**Primer length (nt)**	**Amplicon (bp)**	**Site on genome (GenBank Acc. No. CP034479.1)**
Inv-4	mF: 5**′**-FITC-CTACAAGCATGAAATGGCAGAACAGCGTCG-3**′**	30	188	997943. 997972
	mR: 5**′**-Biotin-CAACCAGATAGGTAGGTAATGGAATGACGA-3**′**	30		998101. 998130

*mF, modified forward primer; mR, modified reverse primer.*

### Addition of a Probe Into the RPA Reaction

**FIGURE 4:**
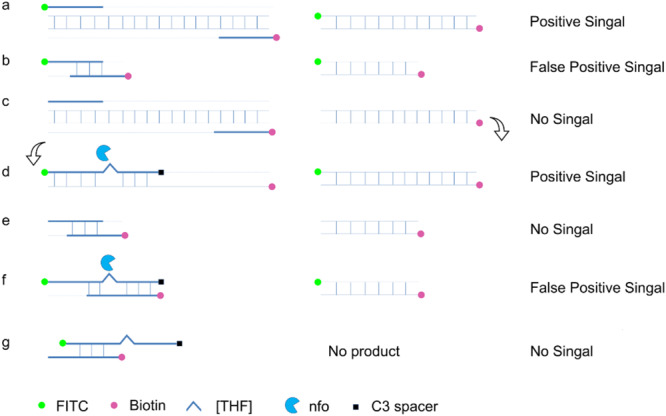
Schematic representation of a specially designed probe that eliminates false-positive signals from primer–dimers. In a typical RPA amplification, both the amplification products **(a)** and the primer–dimers **(b)** can give positive signals. In the modified RPA reaction, amplification of the target DNA from the primer pair does not give the positive signal **(c)**. This amplification product **(c)** goes through another round of amplification guided by the probe to give a positive signal **(d)**. Primer–dimers in the modified RPA reaction do not give the positive signal **(e)**. Primer–probe complexes for most of the time do not give the positive signal. Only in a very rare case that the primer and the probe have a good matching of the bases flanking the tetrahydrofuran ([THF]) site can the complex give a positive signal **(f,g)**. DNA strands are presented as horizontal lines, and the base pairings are indicated as short vertical lines between the DNA strands. Anticipated amplification of the DNA strands is indicated as dotted lines. Labels and modifications on DNA as well as nfo enzyme are indicated with different shapes and colors, with the legends given at the bottom of the figure.

Using a probe in the RPA reaction could increase amplification specificity and reduce primer-dependent artifacts (Figure 4; Piepenburg et al., 2006). In a typical RPA reaction, FITC and biotin are labeled at the 5′ end of each primer. The amplification product has both biotin and FITC labels, while the primer–dimers, if formed, also have the same labels that can give the positive signal (Figures 4A,B). When using a probe in the RPA system and putting the FITC label on the 5′ end of the probe instead of on the forward primer, amplification from the primer pair would only give the product with biotin labeled at one end (Figure 4C). The 3′ end of the probe was labeled with a SpC3 that could block the amplification, and a [THF] site was put in the middle of the probe for nfo cleavage. When the probe pairs with one of the amplification product strands, it is cleaved by nfo enzyme at the [THF] site, and the 3**′** end is freed for elongation to produce the positive signal (Figure 4D). With the usage of the probe, primer–dimers cannot give the signal; partially paired probe–primer would not amplify; only in a very rare case that the probe and the primer are matching multiple bases flanking the [THF] site will there be false-positive products (Figures 4E–G). To keep the chance of a rare case to the lowest, bases matching the reverse primer should be avoided, and the probe concentration should be much lower than the primers.

**FIGURE 5:**
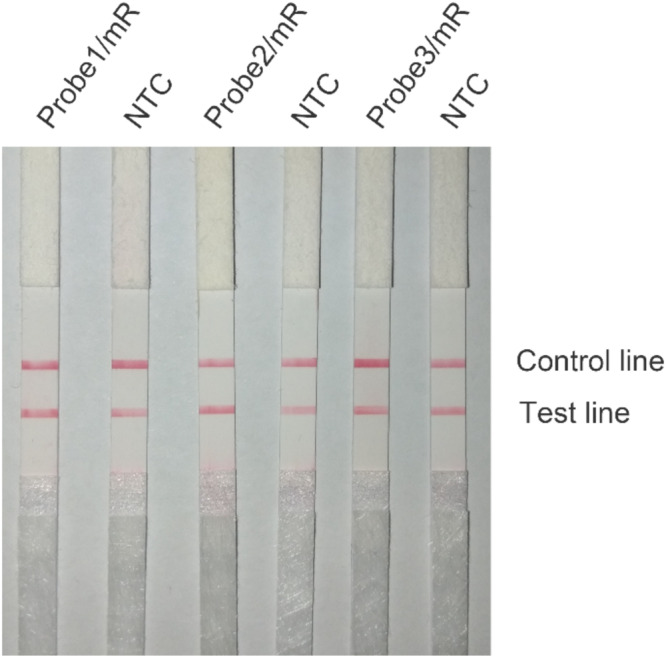
Test of the primer–probe sets on RPA–LFS. The image shows the LFS results of RPA amplifications with different primer–probe sets. The name of each primer–probe set is indicated on the top of each strip. The NTC lanes are the no-template controls of the reactions. The positions of test and control lines are marked on the right of the strip image. The reactions were performed at 40°C for 30 min.

Probe sequences of 46 to 53 nt in length were designed within the targeting fragment of primer pair Inv-4. Parameters were set as there were less than six consecutive bases in the probe that could pair to the primer pair. Eight probe sequences were obtained, and the rating scores of three of them were much higher than the rest (Table 3). The three probes with the highest scores were modified with FITC and SpC3 chemical labels for testing on LFS (Table 4). Results showed that, even with the usage of probes, the false-positive signals were still present (Figure 5).

**TABLE 3:** Design of the probes.

**Probe #**	**Sequence (5′–3′)**	**Primer premier 5 rating**	**Amplicon (bp)**	**Site on genome (GenBank Acc. No. CP034479.1)**
1	5**′**-CTGCTTTCTCTACTTAACAGTGCTCGTTTACGACCTGAATTACTGAT-3**′**	89	109	998022. 998068
2	5**′**-TGCTGCTTTCTCTACTTAACAGTGCTCGTTTACGACCTGAATTACTG-3**′**	89	111	998020. 998066
3	5**′**-TACTTAACAGTGCTCGTTTACGACCTGAATTACTGATTCTGGTACTA-3**′**	88	99	998018. 998064
4	5**′**-TTTACGACCTGAATTACTGATTCTGGTACTAATGGTGATGATCATTT-3**′**	59	83	998048. 998094
5	5**′**-ATTTAATATTAACAGGATACCTATAGTGCTGCTTTCTCTACTTAACA-3**′**	57	137	997994. 998040
6	5**′**-CTGTCTTAATTTAATATTAACAGGATACCTATAGTGCTGCTTTCTCT-3**′**	54	145	997986. 998032
7	5**′**-AGCTGTCTTAATTTAATATTAACAGGATACCTATAGTGCTGCTTTCT-3**′**	54	147	997984. 998030
8	5**′**-AAAGCTGTCTTAATTTAATATTAACAGGATACCTATAGTGCTGCTTT-3**′**	54	149	997982. 998028

**TABLE 4:** Primer probe set Inv-4 sequences and chemical modifications.

**Name**		**Sequence (5′–3′)**	**Amplicon (bp)**
Inv-4	F	5**′**-CTACAAGCATGAAATGGCAGAACAGCGTCG-3**′**	188
	mR	5**′**-Biotin-CAACCAGATAGGTAGGTAATGGAATGACGA-3**′**	
	Probe1	5**′**-FITC-CTGCTTTCTCTACTTAACAGTGCTCGTTTAC[THF]ACCTGAATTACTGAT-SpC3-3**′**	109
	Probe2	5**′**-FITC-TGCTGCTTTCTCTACTTAACAGTGCTCGTTTA[THF]GACCTGAATTACTG-SpC3-3**′**	111
	Probe3	5**′**-FITC-TACTTAACAGTGCTCGTTTACGACCTGAATT[THF]CTGATTCTGGTACTA-SpC3-3**′**	99

*F, forward primer; mR, biotin-modified reverse primer; P, probe.*

### Elimination of False-Positive Signals With Base Mismatches

**FIGURE 6:**
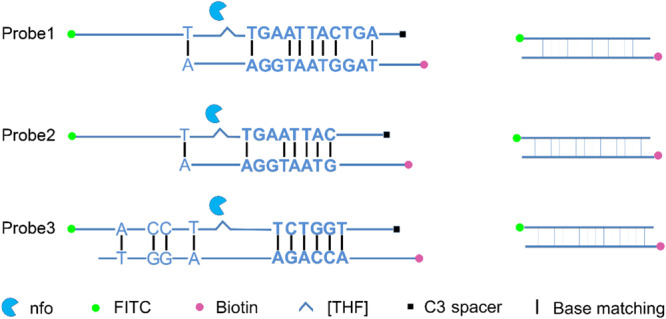
Analysis of the dimer structure between the probe and reverse primer. The reverse primer could form a five-consecutive base matches with Probe1 or Probe2, and a six-consecutive base matches with probe3. Labels and modifications on DNA, nfo, base matching as well as mismatching are indicated with different shapes and colors, with the legends given at the bottom of the figure.

**FIGURE 7:**
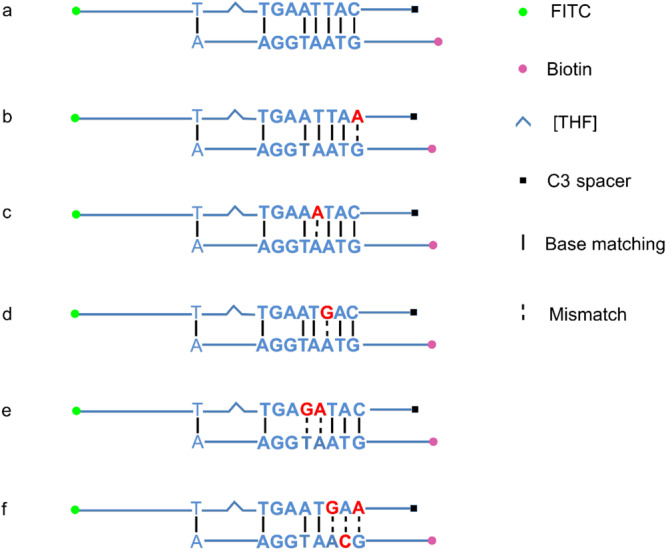
Introduction of mismatches into the primer-probe set. The original primer–probe set probe2/reverse primer had five consecutive matching bases and could form a primer–probe complex that finally gave false-positive signal **(a)**. One base substitution made on the probe at different positions could not disrupt the consecutive base matching and gave false-positive signals **(b–d)**. The probe with two base substitutions still formed a three-consecutive base matching with reverse primer and gave false-positive signal **(e)**. Simultaneous three base substitutions, two on the probe and one on the reverse primer, avoided this matching and gave no product/signal **(f)**. Labels and modifications on DNA, nfo, base matching, as well as mismatching are indicated with different shapes and colors, with the legends given at the bottom of the figure.

**FIGURE 8:**
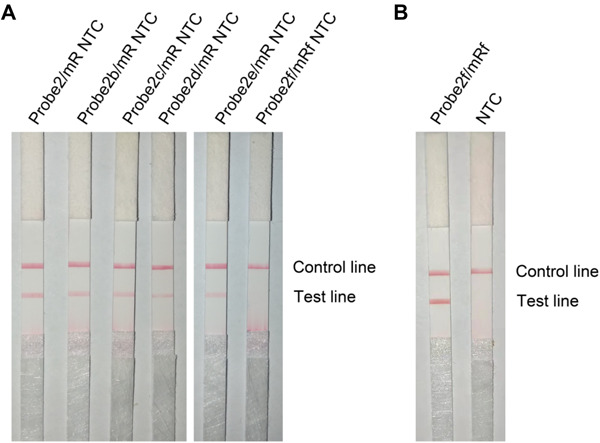
Test of the base-substituted primer–probe sets on RPA–LFS. **(A)** These base-substituted probes and primers were tested for false-positive signals in RPA reaction and LFS detection with no DNA template. **(B)** The optimized primer–probe set Probe2f/mRf was tested for amplification performance in RPA-LFS. The NTC lanes are the no-template controls of the reactions. The name of each primer–probe set is indicated on top of the corresponding strip. The positions of test and control lines are marked on the right of the strip image. The reactions were performed at 40°C for 30 min.

A careful sequence analysis of the three probes and the reverse primer indicated that there were still consecutive base matches between the probes and the primer (Figure 6). Moreover, the base matches were distributed at both sides of the [THF] site, which were able to facilitate the nfo enzyme cutting. This could be the reason why the false-positive signals were still present. Since the RPA system could tolerate some base mismatches on primers without significantly affecting the amplification efficiency (Daher et al., 2015; Liu et al., 2019), base substitutions were systematically introduced into Probe2 and the reverse primer (Figure 7 and Table 5, substituted bases in red). These base-substituted probes and primers were tested for false-positive signals in the RPA reaction and LFS detection with no DNA template. Results indicated that the false-positive signal was eliminated with three mismatches between the probe and the reverse primer (Probe2f/mRf) (Figure 8A). These base substitutions were further confirmed to have no significant effect on the amplification efficiency by observing the band density (Figure 8B).

**TABLE 5:** Base substitutions in Probe2 and the reverse primer.

**Name**	Sequence *(5′–3′)
Probe2b	5**′**-FITC-TGCTGCTTTCTCTACTTAACAGTGCTAGTTTA[THF]GACCTGAATTA**A**TG-SpC3-3**′**
Probe2c	5**′**-FITC-TGCTGCTTTCTCTACTTAACAGTGCTAGTTTA[THF]GACCTGAA**A**TACTG-SpC3-3**′**
Probe2d	5**′**-FITC-TGCTGCTTTCTCTACTTAACAGTGCTAGTTTA[THF]GACCTGAAT**G**ACTG-SpC3-3**′**
Probe2e	5**′**-FITC-TGCTGCTTTCTCTACTTAACAGTGCTAGTTTA[THF]GACCTGA**GA**TACTG-SpC3-3**′**
Probe2f	5**′**-FITC-TGCTGCTTTCTCTACTTAACAGTGCTAGTTTA[THF]GACCTGAAT**G**A**A**TG-SpC3-3**′**
mRf **	5**′**-Biotin-CAACCAGATATGTAGG**C**AATGGAATGACGA-3**′**

*The substituted sites were marked as red and underlined. **mRf, base-substituted biotin-modified reverse primer.

### Detection Performance of the Primer–Probe Set in RPA–LFS

**FIGURE 9:**
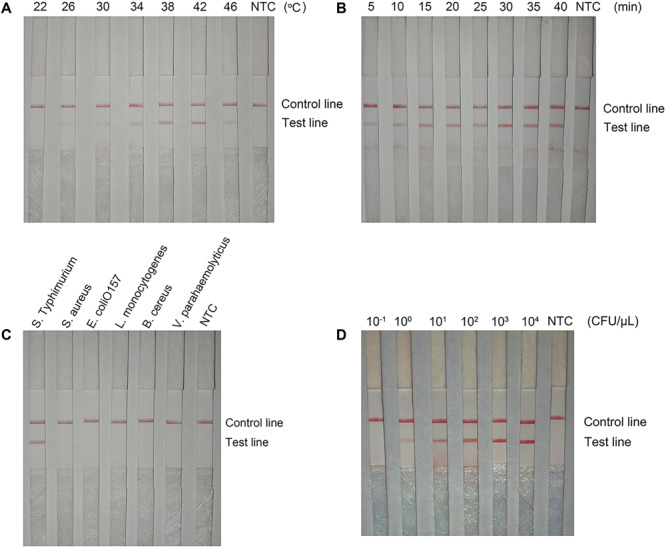
The performance of the optimized primer–probe set in RPA–LFS. **(A)** Optimal reaction temperature of the RPA–LFS test. The image shows the LFS results of RPA amplifications under different temperatures. The temperatures under which the RPA reactions were performed are indicated on top of each strip. The amplification template was *Salmonella enterica* serotype Typhimurium (*S.* Typhimurium). The NTC strip is the no-template control that performed at 37°C. The positions of the control and test lines are indicated on the right of the image. **(B)** Optimal reaction time of the RPA–LFS test. The image shows the LFS results of RPA amplifications with different times. The time for which the RPA reactions were performed are indicated on the top of each strip. The amplification template was *S.* Typhimurium. The NTC strip is the no-template control that performed for 40 min. The positions of the control and test lines are indicated on the right of the image. **(C)** Detection specificity of the optimized primer–probe set. Specificity assay of RPA–LFS was conducted with different bacterial templates. The species of the bacteria are indicated on the top of each strip. The NTC strip is the no-template control. The positions of test and control lines are marked on the right of the strip image. The reactions were performed at 42°C for 30 min. **(D)** Detection limit of the RPA–LFS test. The image shows detection limit of the RPA–LFS system. The image shows the LFS results of RPA amplifications with different CFU of *S.* Typhimurium. The amounts used in the RPA reactions are indicated on top of each strip. The NTC strip is the no-template control. The reactions were performed at 42°C for 15 min. The test line and control lines are marked on the right side of the image.

The detection performance of the primer–probe set (Inv-4-F/Probe2f/mRf) was tested in RPA reaction and LFS reading. The reaction temperature was screened from 22 to 46°C (Figure 9A). The signal band at the test line was visible between 26 and 46°C with the best density at 42°C. The reaction time was tested in the range of from 5 to 40 min (Figure 9B). A weak signal band appeared at 5 min and kept increasing with the extended reaction time. After 30 min, the signal density did not change significantly. The detection specificity was assessed with several other commonly seen pathogenic bacterial species, including *S. aureus*, *E. coli* O157, *L. monocytogenes*, *B. cereus*, and *V. parahaemolyticus*. Results showed that only a positive result was seen in the *S.* Typhimurium culture solution, and all the other bacterial cultures were negative (Figure 9C). To determine the detection limit, a 10-fold series dilution of inactivated *S.* Typhimurium ranging from 10^–1^ to 10^4^ CFU/μl was tested. The results showed a signal band at the test line with 1 CFU/μl, and the signal density increased with the increasing amounts of *S.* Typhimurium (Figure 9D). Thus, the detection limit was 1 CFU/μl.

### Application Simulation of the RPA–LFS Test for *S.* Typhimurium Detection

The RPA–LFS test was applied to *S.* Typhimurium detection in artificially contaminated milk samples, and the detection accuracy was compared with the traditional qPCR method. Fifty milk samples were prepared with eight of them artificially contaminated with *S.* Typhimurium. The 50 samples were randomly numbered and subjected for detection of *S.* Typhimurium with both RPA–LFS and qPCR. All the eight artificially contaminated samples were successfully detected, and the results of the RPA–LFS method were consistent with those from qPCR (Table 6).

**TABLE 6:** Detecting performance of the recombinase polymerase amplification (RPA)–lateral flow strips (LFS) test and quantitative polymerase chain reaction (PCR).

**No.**	**Artificially add *S.* Typhimurium**	**RPA–LFS**	**Quantitative PCR**		**No.**	**Artificially add *S.* Typhimurium**	**RPA–LFS**	**Quantitative PCR**	
			**Result**	**Ct value (*n* = 3)**				**Result**	**Ct value (*n* = 3)**
1	No	−	−	40.00 ± 0.00	26	No	−	−	40.00 ± 0.00
2	No	−	−	40.00 ± 0.00	27	No	−	−	40.00 ± 0.00
3	No	−	−	38.46 ± 0.31	28	No	−	−	40.00 ± 0.00
4	Yes	+	+	21.52 ± 0.07	29	Yes	+	+	17.65 ± 0.05
5	No	−	−	34.96 ± 0.00	30	No	−	−	40.00 ± 0.00
6	No	−	−	40.00 ± 0.00	31	No	−	−	40.00 ± 0.00
7	No	−	−	40.00 ± 0.00	32	No	−	−	40.00 ± 0.00
8	No	−	−	35.72 ± 0.00	33	Yes	+	+	20.59 ± 0.13
9	No	−	−	40.00 ± 0.00	34	No	−	−	40.00 ± 0.00
10	Yes	+	+	17.75 ± 0.06	35	No	−	−	40.00 ± 0.00
11	No	−	−	40.00 ± 0.00	36	No	−	−	40.00 ± 0.00
12	No	−	−	38.77 ± 0.00	37	No	−	−	40.00 ± 0.00
13	No	−	−	40.00 ± 0.00	38	No	−	−	40.00 ± 0.00
14	No	−	−	34.99 ± 0.00	39	No	−	−	40.00 ± 0.00
15	Yes	+	+	21.79 ± 0.39	40	No	−	−	40.00 ± 0.00
16	No	−	−	32.39 ± 0.00	41	No	−	−	40.00 ± 0.00
17	No	−	−	40.00 ± 0.00	42	No	−	−	40.00 ± 0.00
18	Yes	+	+	17.78 ± 0.01	43	No	−	−	36.88 ± 0.00
19	No	−	−	40.00 ± 0.00	44	No	−	−	40.00 ± 0.00
20	No	−	−	40.00 ± 0.00	45	Yes	+	+	20.72 ± 0.05
21	No	−	−	37.86 ± 0.27	46	No	−	−	40.00 ± 0.00
22	No	−	−	40.00 ± 0.00	47	No	−	−	40.00 ± 0.00
23	No	−	−	37.00 ± 2.63	48	Yes	+	+	30.07 ± 0.24
24	No	−	−	40.00 ± 0.00	49	No	−	−	40.00 ± 0.00
25	No	−	−	40.00 ± 0.00	50	No	−	−	40.00 ± 0.00

*“+” means positive. “−” means negative.*

## Discussion

Currently, long food supply chains and extensive cooperations among food-related enterprises have put a big challenge on food safety management. For controlling foodborne pathogenic bacteria, fast, accurate, and simple technologies that can be applied to on-site detections under resource-limited settings are required all the time (Ma et al., 2017). Combining the isothermal RPA reaction with the LFS end-point readout method is a promising solution because of the short reaction time, low isothermal condition, and simple and instrument-free procedure (Wu et al., 2016). However, these advantages come with a non-negligible intrinsic risk of the RPA–LFS combined method, the primer-dependent artifacts. Primer–dimer formation is affected by many factors such as buffer contents, environment temperature, and mixture impurities and is hard to be avoided (Das et al., 1999). Hot-start strategies that can reduce primer–dimer formations in PCR reactions are not applicable to RPA. The LFS method could not distinguish the size of signal-giving molecules and takes every such molecule as a positive signal. Considering the high sensitivity of the method (Miao et al., 2019), a very low amount of primer–dimers is a significant risk that can lead to a false-positive detection.

Careful selection of primer-targeting sequences to avoid consecutive matching bases between primer pairs could be useful to prevent the primer–dimer formation. However, in many cases, the options of targeting sequences were limited because the effective detection biomarkers of the pathogens were restrained usually to the virulence genes (Aher et al., 2012). In the design of primers for the detection of *S.* Typhimurium, we limited the targeting sequences in the two virulence genes, *InvA* and *InvE*, and put strict criteria to maximally avoid the chance of primer–dimer formation. The results indicated that, even with these precautionary measures, the primer pairs still had severe primer–dimer problem (Figures 2, 3). The primer pair Inv-4 did not show the primer–dimer band on the agarose but gave false-positive signal on the strip, indicating a much higher sensitivity to primer-dependent artifacts by the LFS method than the gel electrophoresis.

In addition to the primer pair, using a probe in the RPA reactions could increase the specificity and reduce the primer-dependent artifacts (Dong et al., 2020; Wang et al., 2020). This measure has been proven to be effective in some cases (Kersting et al., 2014). In the case of detecting *S.* Typhimurium, however, the false-positive problem was not solved by just using the probe (Figure 5). Because of the chemical labeling of the probe and the reverse primer, the false-positive signal was coming from the primer–probe complex instead of the primer–dimers. We confirmed that the false-positive signal was indeed from the primer–probe complex by cloning and DNA sequencing. As the probe concentration was much lower than the primers, and the 3′ end of the probe could be only open for elongation with the nfo cleavage, this measure should have reduced the chance of primer–probe complex formation, but the effect was not observed due to the high sensitivity of the LFS method.

The probe sequences were indeed able to pair with the reverse primer to some extent (Figure 6). Since the probe sequences had to be selected within the region defined by the primer pair, perfect probe sequences without any consecutive matching bases to the primer were difficult to find. We utilized the feature that the RPA reaction could tolerate some base mismatches on forward and reverse primers to the template and tried base substitutions on the probe and the reverse primer in the RPA–LFS (Daher et al., 2015; Liu et al., 2019). The 3′ end of the primer was the elongation site, thus bases close to the 3′ end should not be substituted. Substitution of multiple distantly located bases on the probe and the primer should also be avoided to keep the template recognition capacity. Following these criteria, base substitutions were systematically introduced into the primer and probe sequences. When three mismatches between the probe and the reverse primers were introduced, the probe–primer complex formation was completely prevented, and the false-positive signal was eliminated. Meanwhile, the amplification efficiency was not affected significantly by comparing the signal density differences between Probe2/mR line in Figure 5 and Probe2f/mRf line in Figure 8.

Successful elimination of the primer-dependent artifacts led to the establishment of an RPA–LFS combined method for detection of *S.* Typhimurium that was rapid, specific, accurate, and convenient (Figure 9). The method was able to detect as low as 1 CFU/μl of *S.* Typhimurium in the culture without DNA extraction, and the detection was finished within 30 min under an isothermal temperature between 26 and 46°C. The detection sensitivity of RPA–LFS for *S.* Typhimurium was obviously higher than that of PCR and nearly equal to that of the qPCR method, which claimed a detection limit of 10^3^ CFU/ml using pure culture. However, they were usually associated with 2-h reaction preparation time, PCR amplification, and melt curve analysis (Wang et al., 2018). The RPA–LFS method was much more simple and rapid, thus the detection limit of 1 CFU/μl in culture was satisfactory. In an application simulation, randomly contaminated milk samples were 100% accurately detected.

## Conclusion

The primer-dependent artifacts in the RPA–LFS combined method were successfully eliminated using a probe in the RPA reaction and introducing base substitutions in the primer and probe sequences. This provided practical measures for the prevention of false-positive signals from primer–dimers or primer–probe complexes in pathogen detections when using the RPA–LFS method. The measures should enable better application of the RPA–LFS method to solve the problem of on-site foodborne pathogenic bacteria detections under resource-limited settings. Moreover, a rapid, accurate, and simple detection method that is readily applicable to the *S.* Typhimurium detection has been established.

## Data Availability Statement

All datasets generated for this study are included in the article/supplementary material.

## Author Contributions

JD, SG, CL, and JZ designed the research. HW, PZ, XY, XZ, ZZ, and JZ conducted the research. PZ, SG, JD, and JZ wrote the manuscript. JL provided bacterial strains. HW and JZ analyzed the data. JD directed the project. All authors contributed to the article and approved the submitted version.

## Conflict of Interest

The authors declare that the research was conducted in the absence of any commercial or financial relationships that could be construed as a potential conflict of interest.

## Footnotes

1 https://www.ncbi.nlm.nih.gov/tools/primer-blast
